# DRIVING CESSATION AND SOCIAL ISOLATION IN OLDER ADULTS

**DOI:** 10.1093/geroni/igz038.3048

**Published:** 2019-11-08

**Authors:** Weidi Qin, Xiaoling Xiang, Harry O Taylor

**Affiliations:** 1 Case Western Reserve University, Cleveland, Ohio, United States; 2 University of Michigan, Ann Arbor, Michigan, United States; 3 Washington University in St. Louis, St. Louis, Missouri, United States

## Abstract

This study aimed to investigate the impact of driving cessation on social isolation in older adults. Data came from the National Health and Aging Trends Study round 1 through round 6 surveys. Study sample consisted 6,916 Medicare beneficiaries aged 65+ who were eligible drivers at baseline. Social isolation measure was based on social domains concerning marriage, family and friends, church participation, and club participation. The impact of driving cessation on social isolation was assessed using mixed-effects ordered logistic regression and piece-wise regression. In multivariable mixed-effects ordered logistic regression, past-year non-drivers had a two-fold increase in the odds of being in a higher social isolation category (OR=2.1, p<.001). The odds of social isolation were higher among persons aged 85-89 (OR=1.40, p<.001) or 90+ (OR=1.99, p<.001) as compared with those aged 65-69, among men as compared with women (OR=2.54, p<.001), among persons living alone (OR=3.95, p<.001), with elevated depressive symptoms (OR=1.54, p<.001), and with possible (OR=1.39, p<.001) or probable dementia (OR=1.51, p<.001). The odds of social isolation decreased significantly as education and family income levels increased. Functional limitation was associated with higher odds of social isolation. Piecewise regression analysis showed social isolation score increased by 0.08 points (p=.024), indicating short-term impacts of incident driving cessation. Driving cessation is associated with higher risk of social isolation in older adults. Interventions to provide alternative transportation resources and reduce social isolation among older adults may improve public health impact by targeting older adults who recently stopped driving.

